# The effect of city size on the disability rate of the older adults: empirical evidence from CHARLS data

**DOI:** 10.3389/fpubh.2023.1227234

**Published:** 2024-01-05

**Authors:** Huan Liu, Lianjie Wang, Tiantian Hu

**Affiliations:** ^1^School of Social Society, Soochow University, Suzhou, Jiangsu Province, China; ^2^School of Law, Jiangnan University, Suzhou, Jiangsu Province, China; ^3^Shenzhen Futian District Economic Development Promotion Association, Shenzhen, China; ^4^Qiaocheng Consulting (Shenzhen) Co., Ltd, Shenzhen, China

**Keywords:** city size, urban sprawl index, disability, older adults, CHARLS

## Abstract

**Background:**

Exploring the value orientation of city development from the perspective of people’s well-being is key to clarifying the current debate on city size strategies.

**Methods:**

Based on the survey data of the China Health and Retirement Longitudinal Study in two phases, and by using the unbalanced panel-ordered logit model, stepwise analysis method, and KHB method, among others, we discuss the impact of city size on the disability rate of older adults.

**Results:**

The study finds that city size significantly and positively affects the activities of daily living (ADL) of older adults, with odd ratios of 1.3286 (95% CI = [1.082243, 1.631089]), and exhibits significant group heterogeneity in terms of age, registered residence, income, and education level. City size has indeed played an mediation role through the health improvement effect, disease mitigation effect, emotional effect, and employment structure optimization effect, with a cumulative contribution rate of the mediation effect of 35.17%. In addition, the urban sprawl index has a significant moderation role.

**Discussion:**

Robust urban sprawl is conducive to promoting the improvement of the regional health environment, the improvement of the medical security policy, the optimization of the family support policy, and the employment structure, thus helping to reduce the disability rate of older adults.

## 1. Introduction

Under the guidance of the national strategy of actively coping with population aging, the rapid population gathering in cities, on the one hand, meets the inevitable requirements of economic and social development and, on the other hand, provides meaningful opportunities for promoting population health and improving people’s livelihood (1). Since the reform and opening up, and especially since China entered the aging stage in 1999, the country’s aging and disability rates have been expanding annually, while the urbanization rate has also been developing rapidly. According to the data released by the National Bureau of Statistics of China, by 2021, the number of people aged 60 years and above in China had reached 267 million, accounting for 18.9% of the total population; the population aged 65 years and above had reached 200.6 million, accounting for 14.20% of the total population (2), far exceeding the aging threshold of 7%. According to relevant research, China’s current disabled population is estimated to exceed 50 million people (3). In 2000, there were 262 cities at the prefecture level and higher in China, including 90 cities at the prefecture level, with a total population of over 1 million at the end of the year. However, by 2020, the number of cities in China at the prefecture level and above had increased to 297, including 164 cities at the prefecture level and above, with a total population of over 1 million in urban districts at the end of the year, up by 82.2% (2).

The most important feature of the development of the urbanization rate is the continuous expansion of the urban population size, while population agglomeration also brings about the city-size effect, which is conducive to the construction of a city’s public-service facilities and development of livelihood-security policies (4, 5). Throughout the development process of urbanization in China, the policy of “controlling the size of large cities, rationally developing medium-sized cities and actively developing small and medium-sized cities” has been followed for a long time, which makes China’s overall city pattern a decentralized feature of low city concentration and small average size (6). This city-development model not only limits the formation of the optimal structure of city size but also has an important impact on city residents’ living environment. Specifically, the health-impact and disability risk for older adults in the process of urbanization is further intensified. For example, under an excessive degree of urban sprawl, city-size development, which aim at the potential problems derived from China’s city-development path, will not only cause city congestion, a severe shortage of infrastructure, and other problems (7, 8), but it will also lead to a continuous decline in per-capita medical-and health-service resources due to an increase in population concentration, which will affect residents’ health level.

Therefore, targeted research at the theoretical and empirical levels is of great practical significance for optimizing city-size construction and realizing the goal of healthy aging development. For a long time, research on city size has focused on city welfare, wage premium, and residents’ health (9–11), while research from the perspective of older adults’ disability risk has been relatively scarce. The aging trend has become a universal social reality. Most existing studies have been conducted from the perspectives of medicine, long-term care (LTC), and security policies (10, 12). On the one hand, they focus on policy intervention after the occurrence of passive disability risk; on the other hand, they ignore the heterogeneity of social and economic environments and other impacts in the region where older adults live. Therefore, this study attempts to consider city size as the main variable to investigate the impact of city-size construction on older adults’ disability rate and its mechanism; the aim is to provide empirical support for the construction of the optimal city size to improve people’s living environment as well as support for an optimization strategy for the external environment to prevent disability in older adults.

## 2. Literature review

Older adults’ health is multidimensional, and the risk of disability is an important result of the healthy production of older adults; thus, research has been conducted from many perspectives. There have been relatively few direct-relevance studies on health at the city level. Existing studies have mainly analyzed urban welfare and residents’ health effects.

First, the analysis of city well-being includes research on the impact of city size on income, inequality, quality of life (QoL), and “city disease.” Research shows that the high wage premium in large cities under the expansion of city size is a monetary compensation and nominal phenomenon (13), while the increase in city-population size is significantly related to regional income inequality. Although the immigration factor plays an important role (9), its regularity is inconsistent. Some studies have confirmed an “inverted U-shaped” relationship between city size and income inequality (14). In terms of QoL, three main conclusions have been drawn: (1) Establish an evaluation index system to evaluate the quality of city life (15, 16). (2) Based on the spatial equilibrium hypothesis, examine the quality of city life from the perspective of implicit value (17–19). (3) Based on the first two types of measurement methods, evaluate city life quality with city comfort as the core (20, 21).

However, there is no unified conclusion on the influence of city size on QoL. For example, some scholars have found that when the city size is 200,000 ~ 500,000, the residents’ QoL is the highest (22), while others have found no linear relationship between city size and residents’ QoL (23). In terms of “city disease,” most studies show that expanding city size will increase the degree of city pollution, which will affect residents’ health; however, this law is nonlinear (24, 25). Simultaneously, a city’s expansion will cause an overall change in the city’s crime rate (26). In addition, scholars have conducted extensive research on the wage spillovers and labor productivity brought about by city size and have investigated urban welfare under the optimal city size (10, 12, 27).

Second, the impact of city size has been investigated from the perspective of residents’ health. Research has shown that an initial expansion of city size can bring about a scale effect in the short term (11, 28, 29). For example, when a city’s size expands, this promotes regional specialization, labor productivity, and so on, thereby improving the residents’ per-capita income level, helping them improve their material living conditions, and thus promoting their health (1, 9, 30, 31). Meanwhile, the expansion of the city’s size will gradually increase regional medical resources and the government’s medical and health expenditure, help to establish rich and high-quality medical and health resources, improve the residents’ access to medical-and health-service resources, and ultimately improve the residents’ health level (1, 32). In addition, the expansion of city size is accompanied by the “migration effect.” The gradual inflow of many healthy immigrants to large-and medium-sized cities will improve the cities’ overall health (33, 34).

However, some studies show that when a city’s size expands to a certain stage, this will lead to a crowding effect, weakening the residents’ health-improvement effect (7, 8, 11). For example, the expansion of a city’s size leads to an increase in infectious diseases, causing potential health risks and hidden dangers, and it has the risk of deteriorating the quality of the city environment, affecting traffic safety, diet, and other risks and thus the residents’ health level (7, 8, 35–37).

The existing literature on the overall effect of city size is relatively rich; however, research on the health effects of city size on older adults is relatively scarce. In particular, the literature on older adults’ disability risk from the perspective of city expansion is almost non-existent. Although research shows that city size has a significant impact on residents’ health and older adults’ disability risk, scholars have reported inconsistent findings; thus, it is urgent to examine whether the gradual expansion of city size will increase the risk of disability. This is not only a critical path to improving the optimal city size and its supportive measures but also an essential part of the external environmental protection to improve older adults’ disability risk. In addition, although existing studies have investigated the impact mechanism of city expansion on residents’ health, they have not considered the urban-sprawl index as a key indicator, which yields an inaccurate assessment of the core of the impact of city expansion.

In view of the foregoing, the main marginal contributions of this study are as follows. First, from the perspective of research, this study examines the formation mechanism for older adults’ disability risk from the perspective of city-size construction to provide a new perspective for the governance of the risk at the macro level. Second, in terms of research content, this study considers older adults’ disability risk as the main variable, and based on the perspective of the difference in city-size development, we first analyze the impact of city size on older adults’ disability risk. Third, this study explores the potential mechanism through which the impact of city size is transmitted to older adults’ disability risk in terms of health-improvement, disease-mitigation, emotional, and employment-structure optimization effects. Finally, based on existing research, this study considers the urban-sprawl index as a vital inspection indicator to empirically test the actual effect of the urban-sprawl degree regulating city size on older adults’ disability risk to provide support for optimizing city size and improving the macro-security mechanism for older adults’ disability risk.

Regarding the research methods, this study first uses a panel-ordered logit model to empirically examine the impact of city size on older adults’ activities of daily living (ADL). Second, by using the stepwise analysis and KHB (Karlson-Holm-Breen) methods, the significance of the mediation effect of city size on older adults’ ADL and contribution of each mediation variable to the cumulative mediation effect on older adults’ ADL were evaluated.

### 2.1. Research design

The core explanatory variable in this study is older adults’ disability risk. We chose the older adults’ ADL as the agent, specifically measured as follows. We chose six basic daily-life items in the questionnaire to represent the agent, and the specific questions were as follows:

1. “Because of health and memory problems, do you have any difficulty dressing? Dressing includes taking clothes out of a closet, putting them on, buttoning up, and fastening a belt.”
2. “Do you have difficulty bathing or showering because of health and memory problems?”
3. “Because of health and memory problems, do you have any difficulty eating, such as cutting up your food?”
4. “Do you have any difficulty getting into or out of your bed?”
5. “Because of health and memory problems, do you have any difficulties using the toilet, including getting up and down?”
6. “Because of health and memory problems, do you have difficulties controlling urination and defecation? If you use a catheter (conduit) or a pouch alone, you are not considered to have difficulties.”

The corresponding options for answers 1 to 4 are “No, I do not have any difficulty”; “I have difficulty but can still do it”; “yes, I have difficulty and need help”; and “I cannot do it.” Finally, the ADL value was obtained based on the scores for the six items, and specific classification criteria were defined according to the scores. Further details are provided in Table 1. The statistical results show that the proportion of severe disabilities is only 0.53% and, with a reduction in disability level, its proportion in the total sample increases; for example, the proportion of the population with mild disabilities has reached 18.61%.

**Table 1:** Processing and statistics of core variable ADL.

Variable	Definition	Assignment	Observations	Percentage
Activity of Daily Living (ADL)	According to the sum of six essential life items, the total score of 6 points is recorded as 5, indicating health	5	9,323	51.85%
	The total score of 7 ~ 9 points is recorded as 4, indicating mild disability	4	3,346	18.60%
	The total score of 10 ~ 14 points is recorded as 3, indicating moderate disability	3	1,105	6.15%
	The total score of 15 ~ 20 is recorded as 2, indicating serious disability	2	229	1.27%
	The total score of 21 ~ 24 points is recorded as 1, indicating severe disability	1	96	0.53%

According to the calculated ADL index and characteristics that conformed to the ranking variable, we established a panel-ordered logit benchmark test model. The model settings were as follows:

(1) $$\begin{array}{l} O\log\mathrm{it}\left(\mathrm{AD}L_{itc}=1...5\right)=\alpha_{0}+\alpha_{1}\mathrm{City}:Size_{itc}\\ \;+\alpha_{2}X_{itc}+Time_{t}+Id_{i}+\varepsilon_{itc} \end{array}$$

The ADL in Equation (1) is recorded as the ability of the older adult to take care of themselves according to the agency index of disability risk. is a city-size variable. City size was obtained from the population size of the city where the interviewees were located at the time of the survey. During processing, we used logarithmic transformation. The statistical results show that the mean value of the logarithm of city size was 6.1987. In addition, to ensure comparability of the estimated results, we synchronized the statistics for the city categories, specifically dividing them into prefecture-level, provincial capital, and sub-provincial cities and municipalities directly under the central government. Because the sub-provincial cities intersect with the provincial capital and prefecture-level cities, the statistics will be based on the highest level and will not be repeated.

The statistical results show that the proportion of prefecture-level cities in the sample is 73.82%, that of provincial capital cities is 5.79%, and that of sub-provincial cities and municipalities directly under the Central Government is 20.39%. X refers to the control variables. This study mainly controls for individual and family variables such as gender, age, education level, marriage, registered residence, medical insurance, per-capita household income, and macro city-characteristic variables such as city category, regional air quality, per-capita GDP, green coverage, regional financial expenditure, population density, and the urban-sprawl index. Time represents the time-fixed effect, Id is the individual fixed effect, ε is the random interference term, and the symbol and significance of the coefficient, $\alpha_{1}$ , are the key points of the benchmark model. The urban-sprawl index in the benchmark model is calculated as follows:

(2) SIi=1−ΔPPi/PPi0ΔABDi/ABDi0

Accordingly, i refers to the city and SI refers to the urban-sprawl index. The right side of the Equation refers to 1 minus the change value, which is the ratio of the change rate of the regional population size to the change rate of the regional built-up area. In this study, the year 2000 is selected as the base period, where $PP_{i0}$ refers to the regional population at the base period and $\Delta PP_{i0}$ refers to the change value of the regional population compared to that of the base period at the time of investigation; $\mathrm{AB}D_{i0}$ refers to the area of the built-up area in the base period, and $\Delta ABD_{i0}$ refers to the change value of the built-up area in the survey compared to that of the base period. Table 2 shows that the average urban-sprawl index (SI) is 0.8673. Statistical results for the other variables are presented in Table 2. Most variable names are abbreviated, and their specific meanings are provided in the definitions.

**Table 2:** Descriptive statistics.

Variable	Definition	Mean	S.D.	Min	Max
City size	Logarithm of Annual resident population size in urban survey, unit: 10000 persons	6.1987	0.6532	4.3722	8.0397
Gender	Male = 1, Female = 0	0.4918	0.4999	0	1
Age	Age of respondents in the survey year, unit year	68.0625	6.6385	60	115
Edu	The education level (EDU) of respondents in the survey year. The higher the number, the higher the education level	3.0712	1.4911	1	10
Married	Married = 1, Other = 0	0.7968	0.6354	0	1
RR	Registered residence (RR), Urban = 1, Rural = 2	1.6565	0.4749	1	2
MI	If an individual has participated in Medical Insurance (MI) = 1, NO = 0	0.9860	0.1177	0	1
PLC	The respondent is located in a Prefecture Level City (PLC) = 1, NO = 0	0.7382	0.4396	0	1
PC	The respondent is located in the Provincial Capital (PC) = 1, NO = 0	0.0579	0.2335	0	1
SPCM	The respondent is located in the Sub Provincial City& Municipality (SPCM) = 1, NO = 0	0.2039	0.4029	0	1
AQI	Annual average Air Quality Index (AQI) statistical value of the area where the respondents are located in the survey year. The larger the value, the worse the air quality is	79.7010	22.8858	40	206
GPC	Logarithm of GDP Per Capital (GPC) of the respondents in the survey year	10.6802	0.6189	9.3034	12.2909
GC	Logarithm of Green Coverage (GC) of respondents in the survey year	3.6640	0.2326	1.9095	4.2047
FE	The logarithm of Financial Expenditure (FE) of the respondent’s location in the survey year	6.0634	0.7677	2.9178	9.0302
INCOME	Logarithm of household *per capita* income	7.9977	1.6294	0.0000	13.3057
PD	Population Density (PD) of the respondent’s location	501.777	455.923	5.1360	6521.55
SI	Urban sprawl index (SI), the larger the value is, the higher the SI is	0.8637	0.4124	−1.8073	3.9693

In addition, considering the existence of a potential transmission mechanism for the impact of city size on older adults’ disability risk, it is necessary to investigate the potential transmission mechanism of the benchmark test model. According to the stepwise analysis method, the potential transmission mechanism model in this study was set as follows:


(3) $$MI_{itc}=\beta_{0}+\beta_{1}\mathrm{City}:Size_{itc}+\beta_{2}X_{itc}+Time_{t}+Id_{i}+\theta_{itc}$$

(4) $$\begin{array}{l} \mathrm{Ologit}\left(\mathrm{AD}L_{itc}=1...5\right)=\chi_{0}+\chi_{1}\mathrm{City}\_Size_{itc}+\chi_{2}MI_{itc}\\ \;+\chi_{3}X_{itc}+Time_{t}+Id_{i}+\vartheta_{itc} \end{array}$$

**Figure 1:**
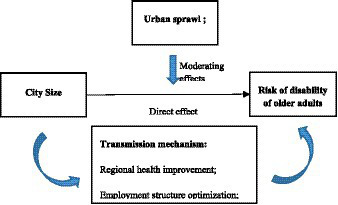
Impact mechanism of city size on disability risk of the older adults.

Equation (3) examines whether city size significantly affects the mediation variable, MI . Equation (4) examines the significance and coefficient changes after introducing intermediate variables into the benchmark model; the significance of the coefficients $\beta_{1}$ , $\chi_{1}$ , and $\chi_{2}$ under Models (3) and (4) is the focus of the mediation effect. If the three are significant, this indicates that a mediation effect must exist, and there is a full mediation. If $\beta_{1}$ is insignificant, this implies that a mediation effect does not exist. If $\beta_{1}$ is significant, and one of $\chi_{1}$ and $\chi_{2}$ is significant, then there is partial mediation. The mechanism diagram for city size and older adults’ disability risk is shown in Figure 1.

### 2.2. Data

The article data were selected from the China Health and Retirement Longitudinal Study (CHARLS) database survey data for (9, 26). The CHARLS survey database covers 122 out of 28 provinces in China (the survey sample does not include Hainan Province, Tibet Autonomous Region, and Ningxia Autonomous Region), and the main survey data are from people aged 45 years and above, which can better reflect the basic characteristics of China’s older adult population. In terms of survey time, the survey data published for each issue were scattered each year. For example, the survey samples in 2018 covered those in 2016, 2017, and 2018. Therefore, the city size information of the current period was determined according to the actual survey year and month of the sample to achieve accurate matching between macro and micro survey data. In this study, the main population was aged 60 years and above. Unbalanced panel data were constructed through data screening and selection, and 17,981 effective samples covering the follow-up survey were obtained.

The descriptive statistics of the core variables of this study, including city size, individual family characteristics, and city characteristics, are shown in Table 2.

## 3. Results

### 3.1. Benchmark test results

First, based on the benchmark model, combined with macro survey data, we empirically examined the impact of city size on the ADL of older adults using the stepwise regression analysis method. Table 3 Models (1)–(3) are the results of controlling different variables, and the results in Model (1) show that city size has a significant positive correlation with the ADL level of older adults; that is, city size can significantly improve the ADL level of older adults. Gender, age, education level, marital status, registered residence, prefecture-level cities, and provincial capital cities in the control variables have significant effects, among which prefecture-level cities (PLC) and provincial capital city (PC) categories are more significant than sub-provincial cities and municipalities (SPCM). Model (2) is the test result of introducing urban macro characteristic variables based on Model (1). The results show that after controlling for the macro characteristic variables of the city, city size still has a significant positive impact on the ADL of older adults.

**Table 3:** Benchmark test results.

Variables	(1)	(2)	(3)	(4)	
				OR value	95% Conf. interval
City size	0.0987^*^ (0.0515)	0.2346^**^ (0.1020)	0.2841^***^ (0.1046)	1.3286^***^ (0.1390)	[1.082243,1.631089]
Gender	−0.1162^*^ (0.0644)	−0.0945 (0.0728)	−0.0927 (0.0732)	0.9115 (0.0667)	[0.789719,1.052026]
Age	−0.0469^***^ (0.0049)	−0.0494^***^ (0.0059)	−0.0492^***^ (0.0060)	0.9519^***^ (0.0057)	[0.940854,0.963163]
EDU	0.0586^***^ (0.0181)	0.0494^**^ (0.0213)	0.0527^**^ (0.0214)	1.0541^**^ (0.0226)	[1.010725,1.099377]
Married	−0.0506^***^ (0.0189)	−0.0516^**^ (0.0212)	−0.0497^**^ (0.0213)	0.9515^**^ (0.0203)	[0.912572,0.992091]
RR	−0.3430^***^ (0.0663)	−0.2274^***^ (0.0867)	−0.2275^***^ (0.0875)	0.7965^***^ (0.0697)	[0.670991,0.945462]
MI	0.1976 (0.1786)	0.0432 (0.2081)	0.0417 (0.2090)	1.0425 (0.2179)	[0.6921566,1.57031]
PLC	−0.2866^***^ (0.1095)	−0.1528 (0.1466)	0.0645 (0.1683)	1.0667 (0.1795)	[0.766924,1.483537]
PC	−0.3202^**^ (0.1466)	−0.3684^**^ (0.1729)	−0.2419 (0.1807)	0.7851 (0.1419)	[0.550930,1.118842]
AQI		−0.0042^**^ (0.0020)	−0.0051^**^ (0.0021)	0.9949^**^ (0.0020)	[0.99088,0.998880]
GPC		0.3080^***^ (0.0751)	0.4017^***^ (0.0817)	1.4943^***^ (0.1221)	[1.27319,1.753782]
GC		0.3811 (0.2568)	0.2544 (0.2599)	1.2897 (0.3352)	[0.774891,2.146452]
FE		−0.0761 (0.0857)	−0.0603 (0.0857)	0.9415 (0.0807)	[0.795863,1.113679]
INCOME		0.0400 (0.0246)	0.0365 (0.0248)	1.0372 (0.0257)	[0.988054,1.088793]
PD		−0.0002^**^ (0.0001)	−0.0002^**^ (0.0001)	0.9998^**^ (0.0001)	[0.999555,0.999946]
SI			−0.2275^***^ (0.0843)	0.7965^***^ (0.0671)	[0.675246,0.939536]
Log likelihood	−13546.896	−10573.538	−10464.055		
Number of obs	11,156	8,590	8,490		
Number of groups	9,302	8,150	8,059		

Standard errors in parentheses, ^***^p < 0.01, ^**^p < 0.05, ^*^p < 0.1. OR, odd ratios.

However, AQI, GDP *per capita* (GPC), and population density (PD) in urban macro characteristics have significant effects on the ADL of older adults. Model (3) further introduces the urban sprawl index (SI) based on Model (2), and the results show that the city size still has a significant positive impact on the ADL of older adults. The SI also has a significant negative impact on older adults’ ADL; that is, the higher the SI, the smaller, the older adults’ ADL, and the higher their disability rate. The SI reflects the ratio of the dynamic change in population to the change in urban built-up areas. The larger the value, the more diffuse the city; therefore, the results in Model (3) show that the larger the city expansion, the worse it is for older adults to improve their ADL. Because the benchmark model uses the panel-ordered logit model, its coefficients do not directly reflect the effect. Therefore, based on Model (3), we synchronously reported the OR value of each variable and its 95% confidence interval (CI), as shown in the results in Model (4) in Table 3.

The results in Model (4) show that the OR value of city size is 1.3286; that is, compared with PC and PLC, the ADL of older adults living in SPCM will be significantly higher, the probability of their ADL is 1.3286 times higher than the former, and the 95% CI is [1.082243, 1.631089]. In addition, the OR value of the SI was 0.7965, and the 95% CI was [0.675246, 0.939536], which means that compared with the low SI group, the ADL of the older adults under the high SI group was only 76.65% of the former, which was significantly worse. This result further shows that SI is not conducive to improving ADL in older adults and even increases the overall disability risk of older adults in the region.

### 3.2. Instrumental-variable processing results

As the benchmark-model test may include some factors (e.g., missing key variables), some missing key variables will affect both city size and older adults’ ADL, resulting in biased estimation results. Meanwhile, there is also an “environmental migration effect” (34); that is, residents will selectively migrate to live in big cities because of the better living environment and public-service facilities there, and people will choose to migrate according to their own health level. The probability of people with poor health quality, especially those with high disability risk, migrating to big cities will be reduced, leading to a two-way impact of city size on older adults’ ADL. The Hausman test results reported in Model (2) of Table 4 show that the *p* value is less than 0.5, which rejects the basic assumption of no difference between the instrumental-variable and benchmark models, indicating that the former method should be used. Therefore, given the estimation error caused by the above endogenous problems, we attempt to deal with the endogenous problems using the instrumental-variable method. From the perspective of existing studies, several instruments deal with variables related to city size. Scholars have chosen both geographical dimensions and historical city sizes (1, 6).

**Table 4:** Estimation results of instrumental variables.

Variables	(1)	(2)	(3)	(4)
	Exogeneity test of instrumental variables	IV estimation (IV = logarithm of city size in 1982)		Confidence interval set method (Twostepweakiv)
		First stage	Second stage	
City size	0.1189(0.2008)		0.0849^***^(0.0282)	[0.0351, 0.1466]
City size_1982	0.1795(0.1868)	0.8003^***^(0.0053)		
Control variable	Control	Control	Control	Control
Time fixed	Control	Control	Control	Control
Regional fixed	Control	Control	Control	Control
Number of obs	8,490	8,490	8,490	8,490
Number of groups	8,059		8,059	
Hausman test		22.38^**^(0.0497)		
R2/Log likelihood	−10463.592		0.0540	

Standard errors in parentheses, ^***^p < 0.01, ^**^p < 0.05, ^*^p < 0.1.

In this study, city size for 122 cities in the 1982 survey sample was used as the instrumental variable for processing. The reason is that city size in 1982 is significantly related to that during the survey period, and city size in 1982 does not directly affect older adults’ ADL at the time of the survey. Thus, theoretically, it is consistent with the selection criteria for instrumental variables. Model (1) of Table 4 is the result of testing the exogenous instrumental variables.

The results show that when the 1982 city size was introduced into the benchmark model, it did not significantly impact older adults’ ADL. This result is consistent with the exogenous assumptions, and it also shows that the instrumental variable does not affect older adults’ ADL in other ways than only indirectly through city size; that is, the instrumental variable is effective. Models (2) and (3) of Table 4 are the results obtained after processing the instrumental variables. The results of the first stage show that city size in 1982 had a significant and positive impact on city size at the time of the survey. The results of the second stage show that after the treatment of the instrumental variables, city size still had a significant and positive impact on older adults’ ADL; that is, the benchmark test results are robust. In addition, to ensure the robustness of the instrumental-variable processing results, based on the exogenous test of the instrumental variables, we also selected the weak instrumental-variable method (Twostepweakiv) for testing, which showed confidence sets (if calculated) based on the 100 points in [−0.199,505, 0.381,228] and a partition cutoff of 5% based on the grid.

Meanwhile, as shown in Model (4) of Table 4, the robust K_ 2sls, LC_2sls, and confidence sets (CS) of AR (Arellano-Bond test) are [0.0351, 0.1466], indicating that the instrumental variable is valid.

### 3.3. Heterogeneity test results

The effect of city size on the ADL (disability risk) of the older adult shows heterogeneity due to group differences. Based on benchmark research, we attempted to examine the group heterogeneity of city size affecting the disability risk of older adults from four aspects: age (AGE), registered residence (RR), income (INCOME), and education level (EDU).

**Figure 2:**
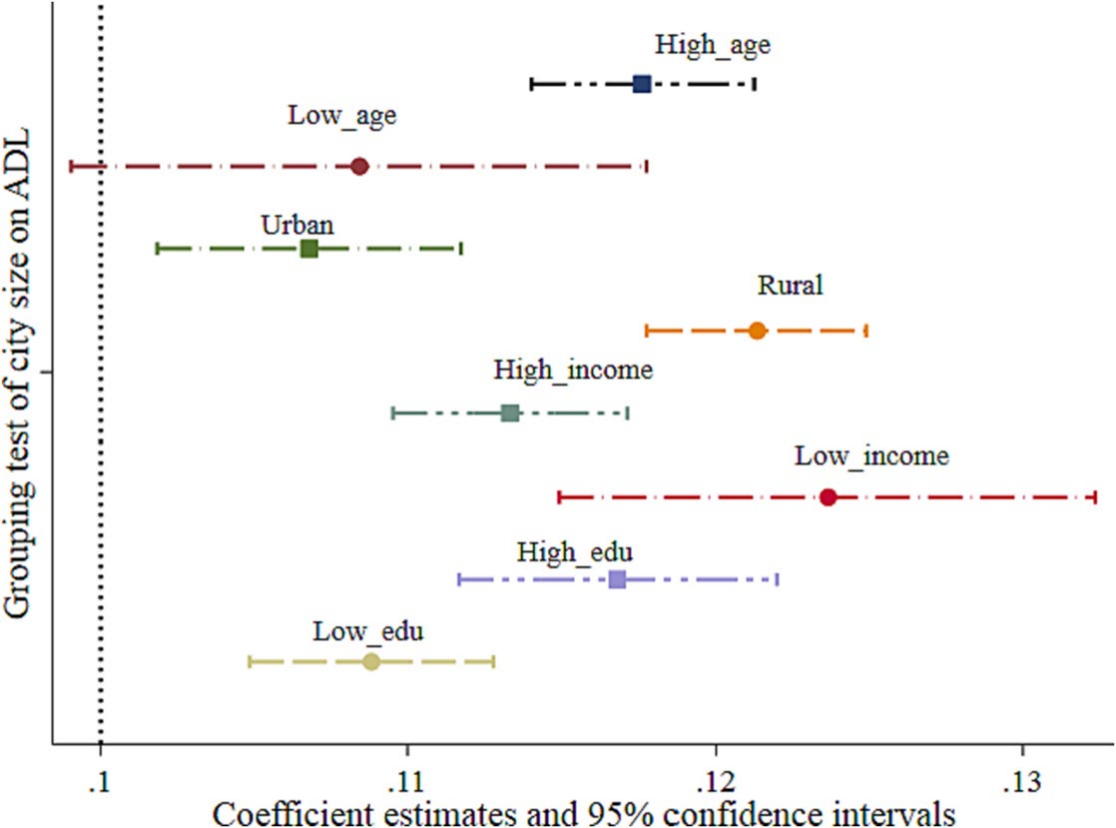
Test results of population heterogeneity.

In terms of AGE, with the rapid development of China’s aging population, the incidence of disability population in China is increasing, and its size is also expanding rapidly. Therefore, it is of great practical significance to study the group heterogeneity in the impact of urban size on the disability risk of older adults from the perspective of age. According to the age division of the welfare policy for the older adult in China, we take 80 years old as the group boundary of the older adults, the younger ones are under 80 years old, and the older ones are over 80 years old. Figure 2 shows the effect of city size and its 95% CI in the test results of population heterogeneity. The results show that, in terms of AGE heterogeneity, the disability risk of older adults aged 80 years and above is more affected by city size than that of young adults. It also shows that with an increase in the age of older adults, their physical functions decline, and when the risk of disability gradually increases, they need more long-term care. In the context of the gradual weakening of family pension security, older adults are affected more by the more perfect long-term care service security system in big cities. They can obtain higher service satisfaction from big cities, thus showing group heterogeneity.

In terms of RR, we mainly divided RR into urban and rural areas according to the characteristics of registered residences of urban and rural populations in China. The empirical test results are presented in Figure 2. It can be seen from Figure 2 that, compared with urban older adults, the disability risk of rural older adults is more affected by city size. With the rapid development of urbanization and population mobility in China, more and more rural older adults are gradually migrating to urban areas under the trend of urbanization and enjoying the benefits of urban public service resources. Especially, in large cities, the radiation of urbanization enables rural residents to obtain more long-term care security service resources, thereby improving their ADL and reducing their disability risk. Under the same circumstances, the disability risk of urban older adults is less affected by the expansion of the city size than that of rural older adults.

In terms of INCOME, we divide household income into five levels: 20% of the highest income, 20% of the upper-middle income, 20% of the middle income, 20% of the lower-middle income, and 20% of the lowest income. In this study, the middle-income and lower-income groups are defined as the low-income group, while the upper-middle-income and highest-income groups are defined as the high-income group. The test results in Figure 2 show that compared with the high-income group, the older adults in the low-income group had a higher impact effect from the expansion of city size. Compared with the high-income group, the low-income group not only has a relative disadvantage in income but also has lower long-term care security. For example, high-income older adults can meet their disability risk security needs by self-purchasing commercial long-term care insurance or hiring domestic workers to provide basic care services. Therefore, the effect of improving self-care ability due to the expansion of city size was lower.

For EDU, we define people with education levels below junior high school as the low education group and those above junior high school as the high education group. This is because the current education level of older adults in China is still low. The test results in Figure 2 show that, compared with the low education group, the older adults in the high education group had a higher ADL effect from city size expansion. Due to the lower information barriers, such as access to basic city service facilities and understanding of long-term care service security policies, the highly educated population can better utilize existing service resources in large cities, thus showing strong group heterogeneity.

### 3.4. Robustness test results

To ensure the robustness of the benchmark test results, we further conducted a robustness test by selecting and replacing the explained variables, control samples, and individual clustering and eliminating follow-up samples. The results are presented in Models (1)–(6) in Table 5. Models (1) and (2) show the test results of using the method of replacing the explained variable. We selected self-assessment health and single physical-disability variables to replace ADL variables; the values of 1–5 indicate that the health status is worsening; in the single physical-disability variable, 1 indicates disability and 0 indicates none. The test results in Models (1) and (2) show that after replacing the explained variables, city size still significantly affects older adults’ self-assessment health and single physical-disability rate; that is, the test results of the basic model are robust, and Model (3) is the result obtained after excluding the abnormal influence of municipal cities. This is because the levels of municipal cities are much higher than those of ordinary prefecture-level cities and their administrative levels are equivalent to those of provinces, thus affecting the estimation results.

**Table 5:** Robustness test results.

Variables	Self_assessment health (1)	Single physical disability (2)	Exclusion of municipalities cities (3)
City size	−0.2832^***^(0.0770)	−0.5223^***^(0.1231)	0.3132^***^(0.1070)
Control variable	Control	Control	Control
Time& regional fixed	Control	Control	Control
Log likelihood	−12970.377	−2725.2601	−10199.887
Number of obs	8,490	5,291	8,238
Number of ID	8,059	4,993	7,822
Variables	Only PLC are considered (4)	Clustering individuals (5)	Excluding follow-up samples (6)
City size	0.2208^*^(0.1195)	0.2841^***^(0.1066)	0.1878^***^(0.0671)
Control variable	Control	Control	Control
Time& regional fixed	Control	Control	Control
Log likelihood	−8837.6839	−10464.055	−9818.4651
Number of obs	7,091	8,490	7,929
Number of ID	6,762	8,059	-

Standard errors in parentheses, ^***^p < 0.01, ^**^p < 0.05, ^*^p < 0.1.

However, the results remain stable after excluding the observations from municipal cities, and Model (4) is the test result after controlling for the PLC sample. Without considering other types of cities, PLC city size still significantly impacts older adults’ ADL. Model (5) is the test result after clustering individuals, while Model (6) is the result after excluding follow-up samples. Both models show that the results of the significant impact of city size on older adults’ ADL are robust.

### 3.5. Test for the potential impact mechanism

The benchmark test examined only the direct impact of city size on older adults’ ADL, while the potential mechanism of the impact requires further discussion. At a micro level, older adults’ ADL results from dynamic changes in their health; that is, in most cases, chronic or major diseases cause the risk of disability. Therefore, we explore the potential mediating mechanism of the effect of city size from a health perspective, that is, to investigate older adults’ disease-relief and emotional effects. At a macro level, a change in city size will bring about macro impact effects, such as the number of doctors per thousand in the city and the number of employees in the secondary industry in the region, especially the NEMI. Therefore, we examine the macro mediation effect of city size in terms of two aspects: the health-improvement and industrial-structure optimization effects. Among them, the health-improvement effect selects the DPP in a region as the proxy variable, TWPR is selected as the agent for the disease-remission effect, DOD is chosen as the proxy for the emotional effect, and the industrial-structure optimization effect selects the logarithm of the NEMI as the agent.

First, based on the selection of the above four mediation variables, we examined whether mediation effects existed. The results are presented in Table 6. The results in Table 6 Model (1) show that in the first stage, city size has a significant and negative impact on the DPP, TWPR, and NEMI in a city and a significant positive impact on older adults’ DOD. This result indicates that city size affects the above mediation variables. In Table 6, the results of the second stage show that after controlling for the relevant mediation variables in the model, city size and each mediation variable play significant roles in simultaneously influencing older adults’ ADL, indicating that a mediation effect exists. Meanwhile, when the health-improvement effect is considered, the impact effect of city size increases from 0.2841 to 0.2883.

**Table 6:** Mechanism test.

Variables	First stage			
	DPP (1)	TWPR (2)	DOD (3)	NEMI (4)
City size	−0.1472^***^(0.0247)	−0.2441^***^(0.0624)	0.2248^***^(0.0732)	−0.2554^***^(0.0592)
Control variable	Control	Control	Control	Control
Time/regional fixed	Control	Control	Control	Control
R^2^/Log likelihood	0.2480	−11990.143	−9207.549	0.0970
Number of obs	8,590	8,590	8,590	8,490
Number of groups	8,151	8,151	8,151	8,059

Variables	Second stage			
	DPP (5)	TWPR (6)	DOD (7)	NEMI (8)
City size	0.2883^***^(0.1048)	0.1960^*^(0.1005)	0.2498^**^(0.1097)	0.2684^**^(0.1046)
Mediation variable	−0.1394^+^(0.1173)	−0.4221^***^(0.0312)	0.7264^***^(0.0574)	−0.0515^***^(0.0171)
Control variable	Control	Control	Control	Control
Time/regional fixed	Control	Control	Control	Control
R^2^/Log likelihood	−10463.346	−10325.35	−10333.758	−10459.451
Number of obs	8,490	8,490	8,490	8,490
Number of groups	8,059	8,059	8,059	8,059

Standard errors in parentheses, ^***^p < 0.01, ^**^p < 0.05, ^*^p < 0.1, ^+^< 0.15. DPP, Doctors per 1,000 people; TWPR, two-week prevalence rate; DOD, degree of depression; NEMI, number of employees in the mining industry.

However, when the disease-mitigation, emotional, and employment-structure optimization effects are considered, the impact effect of city size decreases to 0.1960, 0.2498, and 0.2684, respectively, which further confirms the existence of disease-mitigation, emotional, and employment-structure optimization effects.

Second, to further investigate whether a mediation effect exists and its contribution, we used the KHB method. The results are presented in Table 7, which shows that the four mediation variables are significant; that is, a mediation effect exists. Meanwhile, according to the estimated results of the model, the total contribution rate of the four mediation variables to the city-size effect was 35.17%; that is, the total mediation effect was 35.17%. Among them, the emotional and employment-structure optimization effects were the two most critical, with contribution rates of 11.78% and 17.77%, respectively, while that of the health-improvement effect was only 0.77%. Overall, 35.17% of the impact of city size on older adults’ ADL was transmitted through the health-improvement, disease-mitigation, emotional, and employment-structure optimization effects.

**Table 7:** Mediation effect test results.

Variables	Mediation effect		Contribution	Symbol
	Coef	Std_Err	P_Reduced	
DPP	−0.0011^*^	0.0008	0.77%	−
TWPR	0.0071^***^	0.0020	4.85%	+
DOD	0.0172^***^	0.0045	11.78%	+
NEMI	−0.0259^***^	0.0044	17.77%	−
Total			35.17%	

^***^p < 0.01, ^**^p < 0.05, ^*^p < 0.1. HT, health technicians; TWPR, two-week prevalence rate; DOD, degree of depression; AQI, air quality index; GPC, GDP per capita; RR, registered residence.

Finally, due to the existence of the urban-sprawl effect, to effectively analyze the role of optimal city size, we further investigated the moderation effect of the urban-sprawl index and city air-pollution intensity. The results are presented in Table 8. Models (1) and (2) in Table 8 control for city size × SI and city size × AQI_100 days, respectively, while Model (3) controls for both. The results show that city size × SI and city size × AQI_ 100 days all show significant and negative effects; that is, when the city size reaches a certain level, the higher the urban sprawl and the more severe the city-pollution intensity, the more significant the positive impact of city size on older adults’ ADL, thus inhibiting the health and welfare effects of city size. These findings further show that the larger a city, the better it is; however, it requires a reasonable layout and healthy development. Meanwhile, the city must be alert to the risk of air-quality deterioration during its expansion.

**Table 8:** Moderation effect of urban sprawl and air pollution.

Variables	(1)	(2)	(3)	
City size	0.3261^***^(0.1065)	0.3033^***^(0.1085)	0.3973^***^(0.1134)	[0.1750,0.6197]
City size×SI	−0.0432^***^(0.0150)		−0.0406^***^(0.0150)	[−0.0006,0.00001]
City size×AQI_100day		−0.0003^*^(0.0001)	−0.0003^*^(0.0001)	[−0.0701,-0.01112]
Control variable	Control	Control	Control	
Time/regional fixed	Control	Control	Control	
Log likelihood	−10463.5	−10571.653	−10461.647	
Number of obs	8,490	8,590	8,490	
Number of groups	8,059	8,151	8,059	

Standard errors in parentheses,^***^p < 0.01, ^**^p < 0.05, ^*^p < 0.1. AQI_ 100 day refers to the cumulative number of days in a year when the AQI value of the city in which the respondent was surveyed was greater than 100. The larger the value, the higher the pollution intensity.

## 4. Discussion

For a long time, research on older adults’ disability risk has focused on the medical and social aspects; that is, a natural decline in physical function, major diseases, and social participation, among others, are important incentives for disability risk in older adults, while research from the macro perspective has been relatively scarce. Based on the perspective of city construction, this study discusses the effect of city size on older adults’ disability risk, which not only expands the health effect of city construction but also provides a reliable macro perspective for the governance of disability risk in older adults through an empirical study of continuously tracked survey data. This study finds that city size significantly improves older adults’ ADL and reduces their disability risk, and older adults’ ADL are significantly better in large cities than in small ones.

Numerous studies have confirmed that better public-health service resources and per-capita income in large cities can significantly affect the residents’ QoL, thereby improving their health level (38–41). The reduced effect of city size on older adults’ disability risk confirms this conclusion. Simultaneously, this study conducts an in-depth analysis of the transmission mechanism of the impact of city size on older adults’ ADL. The results reveal that city size has strong health-improvement, disease-mitigation, emotional, and employment-structure optimization effects. These mediation effects are responsible for the potential effect of city size on older adults’ health level and then transmit the impact to older adults’ ADL; the cumulative contribution to the total effect reached 35.17%.

However, does this mean that the larger the city, the better older adults’ ADL? The answer is no. The effective development of cities is an integral part of supporting them in promoting older adults’ health, particularly in regulating the risk of disability. We also conducted an empirical test on this moderation mechanism, and the results showed that when city size reached a certain level, an increase in urban sprawl decreased the impact of city size on older adults’ ADL. The fundamental reason is that disordered growth in a city’s size rapidly increases the city’s built-up area and population.

Meanwhile, older adults’ care services, public-health services, and other infrastructure or resources that are closely related to the residents’ lives do not simultaneously increase, which leads to the negative adjustment of the impact of city size by the degree of urban sprawl (39, 42, 43). This further shows that in the process of the rapid development of a city’s size, to reach the optimal city size, the government must formulate a compact city-size development plan at the macro level that is conducive to gradually spreading public service outward to meet the needs of more social residents. In addition, concerning the regulation effect, the air-pollution intensity due to an excessive expansion of city size will also be increased and further transferred to the comprehensive impact on the residents’ health level, thus gradually decreasing the impact of city expansion on older adults’ ADL. From the design of the urban-sprawl index in this study, when a city’s population growth is lower than the expansion rate of the city’s built-up area, the urban-sprawl degree will rise, which will negatively impact older adults’ ADL. For such large cities, as they have not yet reached the optimal city size, according to the measurement standard for urban sprawl, when the overall population size gradually increases, the decline in older adults’ ADL caused by high urban sprawl will be reversed. The above analysis on urban sprawl and city scale will be a critical path to optimizing the development of city size and an important direction for developing public services under the condition of improving city size.

The results of this study not only show that city size has a significant impact on older adults’ disability risk but also show a heterogeneous impact due to age, registered residence, income, education, and other factors. Based on the direct effect of city size, we also investigated the indirect effect of city size on older adults’ ADL. We found that 35.17% of the overall effect of city size on older adults’ ADL was achieved through the health-improvement, disease-mitigation, emotional, and employment-structure optimization effects. The conclusions of this study enrich the literature on the effect of city size on older adults’ ADL and its potential transmission mechanism and provide the necessary support for the optimal development of city size and the supply of public services.

Nevertheless, this study has some limitations. First, city size is represented by a city’s population size. However, we do not extensively discuss other reasonable proxy indicators for city size, which focuses the research conclusions more on the relationship between population-size changes and built-up areas, ignoring the relevant measurement indicators for public infrastructure and service supply. Second, although we discuss the effect of urban-sprawl adjustment on older adults’ ADL, we do not measure and calculate the specific criteria for the optimal city size and how it should coordinate the relationships between the built-up area, population, and public-service infrastructure construction. Moreover, this is a key direction for future research; that is, from the perspective of city planning, to analyze the transmission path between the optimal development of city size, the social residents’ health quality, and older adults’ ADL.

In addition, the research conclusions have the following implications. First, while a city is developing, local governments must synchronously and reasonably plan the overall city infrastructure and public services to meet all residents’ social service needs, especially older adults’ health-security needs. Second, the extent of urban sprawl must be controlled within a reasonable range and cannot be blindly pursued. Government departments should steadily promote the overall coordinated development process. Third, with the increasing risk of population aging and disability, local governments should pay attention not only to the logic of macro governance policies but also to the coordination between city planning and regional security policies and promote the development of valid security policies and aging governance programs that adapt to the expansion of population size.

## 5. Conclusion

Using survey data, we empirically tested the impact of city size on older adults’ ADL and its effects. Therefore, the conclusions of this study are as follows. First, the change in city size has an important impact on older adults’ disability risk, and the greater the city size, the lower the older adults’ disability risk. Second, the influence of city size on the disability risk of older adults shows evident heterogeneity in terms of age, registered residence, income, and education level. Among them, older adults with rural, higher age, lower income and higher education can obtain more disability protection from city development. Third, city size is transferred to the impact on the disability risk of older adults through health improvement, disease mitigation, and emotional and employment structure optimization effects. Finally, the steady development of city size is conducive to improving the ability of older adults’ ADL in the region, but the random expansion and disordered development of city size will be counterproductive. It not only brings about changes in the intensity of air pollution but also restricts the positive effects of the development of city size, thus reducing its direct and indirect effects on the disability risk of older adults.

## Data availability statement

The original contributions presented in the study are included in the article/supplementary material, further inquiries can be directed to the corresponding author.

## Author contributions

HL and LJW drafted and revised it critically for important intellectual content and approved the version to be published and carry out language retouching, modification. TH made a substantial contribution to the concept and design of the work, interpretation of data, and drafted the article. All authors contributed to the article and approved the submitted version.
